# Predictors of Medical Students’ Views toward Research: Insights from a Cross-Cultural Study among Portuguese-Speaking Countries

**DOI:** 10.3390/healthcare10020336

**Published:** 2022-02-10

**Authors:** Gustavo Correia, Margarida Pereira, Andreia Gomes, Maria do Rosário Bragança, Silke Weber, Maria Amélia Ferreira, Laura Ribeiro

**Affiliations:** 1 Department of Public Health and Forensic Sciences, and Medical Education, Faculty of Medicine, University of Porto, 4200-319 Porto, Portugal; g_b_correia@hotmail.com (G.C.); mmiguel06@gmail.com (M.P.); andreiasagomes@gmail.com (A.G.); mameliaferreira@med.up.pt (M.A.F.); 2 Centre for Research on Pandemics & Society, Oslo Metropolitan University, 0167 Oslo, Norway; 3 Faculty of Medicine, University of Katyavala Bwila (FMUKB), Benguela 1725, Angola; rosario.braganca@ciencia.ao; 4 Faculty of Medicine, University Agostinho Neto, Luanda 64346, Angola; 5 Botucatu Medical School, São Paulo University, Botucatu 05508-220, Brazil; silke.weber@unesp.br; 6 I3S-Instituto de Investigação e Inovação em Saúde, Universidade do Porto, 4200-135 Porto, Portugal

**Keywords:** evidence-based medicine, cross-cultural study, medical students, physician-scientists, undergraduate research

## Abstract

Developing the skills and interest in scientific research of medical students is crucial to ensuring effective healthcare systems. As such, in this study, we aimed to assess Portuguese-speaking medical students’ attitudes and perceptions toward scientific research and clinical practice, and how they are influenced by individual characteristics and regional indicators. A total of 455 first-year students from three medical schools in three countries (Portugal, Brazil, and Angola) participated in this study by completing a questionnaire. Portuguese students attributed the most importance to scientific skills and were the most confident in their ability to perform these skills. Angolan students were the most motivated to perform research and integrate it into the curriculum, despite having the most negative attitudes and perceiving themselves as having less ability to perform scientific skills. Brazilian students had the least positive attitudes toward science. In Portugal, attitudes depended on gross domestic product (GDP), while in Angola, they were influenced by the type of secondary school attendance. Portuguese students’ perceptions of scientific skills were related to sex, GDP, type of secondary school, and participation in research. In Brazilian and Angolan students, perceptions were associated with age and research participation, respectively. The findings support the need to promote skills and positive attitudes toward scientific research in future physicians, fostering physician-scientists and improving patient care.

## 1. Introduction

### 1.1. Medical Education and Undergraduate Research

Since Flexner’s 1910 report [1], the need to comprehensively integrate the principles of scientific research into medical curricula has been advocated as a vehicle to improve healthcare system quality [2,3,4,5]. However, such integration is still poor in medical curricula [6,7], contributing to the exacerbation of the decline in physicians, physician-scientists [8,9,10], and medical students interested or already involved in research worldwide [11,12]. A physician-scientist is an MD who conducts clinical research as their main professional activity, directly applying their findings to patient care and disease treatment [8,9,13].

Active scientific education promotes the full development of medical students and enhances critical appraisal and problem-solving skills, which are needed for excellent clinical practice [14,15,16]. Medical students who undertake scientific research develop better use and knowledge of evidence-based medicine, and are more likely to contribute to that evidence with their own research [7,17] and to be accepted for residences and fellowship programs of their choice [18], becoming excellent physicians [3,19]. Additionally, scientific research helps medical students to develop creativity, leadership [2], critical [20] and communication skills [15], as well as spreading, evolving and applying new medical knowledge [16,21,22].

Evidence-based medicine involves the use of epidemiological knowledge while developing and applying research, clinical and public health expertise, and the latest findings in clinical practice and health programs and policies [23]. This allows the definition of generic measures against imminent health-related issues, whereas clinical specialists, guided by these measures, act at the individual patient care level, ensuring that several areas of expertise, such as fundamental and clinical epidemiology, qualitative and quantitative research work, critical appraisal of medical literature, basic medical science, clinical know-how, and health economics, are put to good use for patient and population health [23,24].

Despite all appointed benefits, physicians are often forced to choose between medical research and practice, finding it difficult to reconcile both activities due to the cost [8], time [15,25], and intellectual demands [26]. Therefore, from an early stage in their career, medical students should be exposed to scientific research, broadening their scientific training, as a method to attract them to academic medicine [7,12,27]. The lack of proper research role models and mentorship [28,29], pertinent curricular research experiences, proper linkage between students and researchers and/or research environments [4,19], and adequate teaching of basic principles of scientific research in medical schools [30] lead medical students to discard a research career at an early stage in their academic education.

At this level, many world-renowned medical schools, especially in Europe [31,32] and North America [16,33,34], have already integrated mandatory undergraduate research experiences into their curriculum.

### 1.2. Undergraduate Medical Research Worldwide: The Case of Three Portuguese-Speaking Countries

Worldwide, medical schools are striving to meet essential requirements and core competences that all medical students must develop to become excellent physicians [3,35]. A major component of this is the acquisition of the scientific skills that crucial for a full understanding of medical thinking and process [35].

Portuguese-speaking African countries’ medical schools are adjusting their degrees and qualifications according to European reforms [36,37], despite lacking the resources and defined quality parameters key to promoting core medical competences [36,38,39]. Angola has been making a serious effort toward progress by creating, reforming, and solidifying its (infra)structures and human resources [38,40]; yet, investments are far from meeting demands [40,41]. More investments in education prior to medical school, medical school teachers’ preparation, and actual Angolan medical schools are required to accomplish cost-effective changes in medical education and to ensure the quality of health resources [38,39]. This might foster medical students’ interest in basic science and its development [38], essential for evidence-based practice [17], and help tackle the problem of the lack of physicians [39,42] and physician-scientists [10] in African countries.

Many other medical schools in developing countries experience similar lacks of adequate resources and means to ensure quality [3,7,39]. In Brazil, due to the indiscriminate establishment of new medical schools since the year 2000, efforts to enhance the quality of medical education appear to have been compromised [3], despite this being crucial to the provision quality health services [43]. Nowadays, many Brazilian medical schools have integrated undergraduate research into the curriculum, and others are debating its importance in medical courses. However, students still complain about the lack of institutional support of undergraduate medical research [44,45].

The Bologna process, in 2007, prompted a reformulation of Portuguese medical courses, accompanied by an attempt to integrate scientific research into the undergraduate medical curriculum through compulsory graduation theses [37]. Portuguese medical schools are cutting-edge learning, research, and healthcare institutions, which can assist Portuguese-speaking medical schools with their modernization process by sharing their experience [36].

### 1.3. Skills, Attitudes, and Perceptions of Medical Students toward Science and Scientific Research

Medical education has the immediate goal of developing the core professional skills, attitudes, and behaviors of future physicians [46]. A skill is anything that can be taught, practiced, and mastered in which one might become proficient. Research skills include the ability to optimize the learning process, searching for knowledge by establishing facts, considering new ideas, and formulating new theories that might allow knowledge development [19], including about best practices [2]. Organization skills involve the ability to best use time and effort in both work and personal aspects of life [47]. Communication skills comprise the ability to effectively communicate with patients [48], using verbal and nonverbal elements [49], as well as the ability to efficiently communicate research results to scientific communities [50], where proper data presentation and scientific writing are vital, including in the development of young scientists [51].

A positive attitude toward science and scientific research is a key element of contemporary medical education [6,7], and should be nourished from the beginning of medical school [11], as it enhances critical reasoning and assessment skills throughout the learning of evidence-based medicine [6]. However, little information exists concerning the attitudes and perceptions of medical students toward science and scientific research [6,28] or how they can be affected and, ultimately, modified [6,46]. Additionally, evidence on Angolan and Brazilian medical students’ attitudes and perceptions toward science is scarce or nonexistent [45].

Attitudes are defined as context-dependent and hardly mutable predispositions toward a positive or negative response to an idea [46]. Perceptions are defined as processes of recognition, interpretation, and response to information and stimuli [52]. Generally, attitudes concerning scientific research and future research career choices are shaped by previous scientific research training [53], being directly correlated with scientific methodology instruction in medical schools [6,46], by students’ sex [4,54], academic year [34,55], and social, cultural, and academic environment stimuli relating to scientific research [34,56]. The lack of success in developing positive attitudes may further exclude medical students from research careers and projects [53,57], exacerbating the gap between the laboratory bench and patients’ bedside (“bench-to-bedside”) [58]. Ultimately, assessing undergraduate medical students’ attitudes toward science and scientific research would be a helpful element in guiding students toward academic medicine, whether basic medical research or even academic clinics [6], as practice leads medical science to further new challenges [47].

### 1.4. Research Objectives

In this study, we aimed to assess the attitudes of first-year medical students in three Portuguese-speaking countries toward science and research, motivation to perform research during medical school, importance attributed to scientific skills for clinical practice, and self-perceived ability to perform scientific skills, and how these are influenced by individual (e.g., sex, age, and type and place of secondary school attendance) and regional indicators (economic, geodemographic, educational, and public health). Therefore, this study was guided by two research questions: (1) What are first-year medical students’ attitudes, motivation, and perceptions toward science and research in Portugal, Brazil, and Angola? (2) What are their individual and contextual predictors?

## 2. Materials and Methods

This cross-cultural study involved three medical schools in three Portuguese-speaking countries, none of them having structured research programs directly incorporated into their undergraduate curriculum. The Faculty of Medicine of the University of Porto (FMUP), Porto, Portugal, has its roots in the 19th century and has a total of around 3000 students enrolled in its undergraduate and postgraduate courses. FMUP provides an Integrated Master’s Degree in Medicine, which has one of the highest admission grade point averages (GPAs) of Portuguese faculties [59]. The Faculty of Medicine Botucatu/São Paulo State University (FMB/Unesp), São Paulo, Brazil, was officially established in 1976, although its roots date back to 1963. It provides a degree in Human Medicine and a degree in Nursing with around 120 places per call in total, as well as other postgraduate programs [60]. The Faculty of Medicine of the University of Katyavala Bwila (FMUKB), Benguela, Angola, was established in 2009, though its medical course initiated in 2008, with around 61 annual places [61].

### 2.1. Data Collection

At the beginning of the school year, all medical students enrolled in the first year at FMUP, FMB/Unesp, and FMUKB voluntarily and nonanonymously self-administered the Importance of Scientific Skills for Clinical Practice (ISS4CP) questionnaire [55], a reliable scale (α = 0.69–0.81). There was a one-year gap in data collection between the three schools, being used a cross-sectional design. In total, 372 eligible students, 234 from Portugal, 75 from Brazil, and 63 from Angola, participated in this study, with a response rate of approximately 85%, 83%, and 96%, respectively.

The questionnaire was made available to students in Portuguese and consisted of 4 domains: (1) general information, including age, sex, type of secondary school attendance, place of residence, and previous participation in research projects; (2) attitudes toward science and scientific research, comprising positive and negative attitudes and predisposition to integrate research into the medical curriculum (integration); (3) motivation to perform research during the medical course (motivation); and (4) perception of medical students both of the importance of scientific skills, namely, research, organization, and communication skills, in clinical practice (clinical practice skills) and their own ability to perform them (ability). Research skills comprised literature searching, coping with information technology, data analysis, and English proficiency; communication skills included writing, oral, and visual communication; and organization skills assessed team work, time management, problem-solving, and improving learning abilities. The ISS4CP domains, comprising a total of 28 items, were assessed using 4-point Likert scales, all inverted for the purpose of this study, ranging from 1 (total disagreement, low, not important, or bad) to 4 (total agreement, very high, very important, or good). The response average was computed to calculate the score of each item and its interpretation was straightforward.

We used the legal administrative divisions for every region to determine differences between student populations. Accordingly, for Portugal, we used the Nomenclature of Territorial Units for Statistics II (NUTS II), which divides the country in 5 regions [62]; for Brazil, we used the Federal Units (FU) system, dividing the country in 27 regions [63]; and for Angola, we used the Provinces system, dividing the country in 18 regions [64]. In this study, regions corresponding to the place of completion of secondary education of less than five students were excluded. The regions considered for each country are identified in Table 1. In Portugal, Great Porto is also a northern region, although of additional importance, since 44.44% of FMUP students completed their secondary education in that region. Additionally, Great Porto represents 63.03% of the north’s population in this sample. Thus, first-year medical students from Great Porto were analyzed independently and the remaining northern regions were renamed North without Great Porto (North w/out GP).

The impact of economic differences in the attitudes and perceptions of different populations of first-year medical students was evaluated using the gross domestic product (GDP) per capita of the same year of the enquiries, or the closest available, which was obtained for the regions of each country [65,66,67] and used as an indicator [68]. Angolan and Brazilian GDPs were converted to euros to match the Portuguese GDP. The geodemographic impact [69] was assessed by population density (number of total inhabitants per area in kilometers square). For the total population living in the regions in each country, the latest and closest census’ results [70,71,72,73] were used. Regional literacy rate for people aged 15 years and above was assessed to estimate educational influences on the attitudes and perceptions of the students. 

Although Portugal [70] and Brazil [73,74] have literacy rates for the full extent of the population, both at the regional and national levels, Angolan data [72] availability is restricted to this age group. The regional basic sanitation access rate was considered as a proxy for infant, child [75,76], and maternal mortality rates [75] to evaluate public health effects on student attitudes and perceptions. Once again, though Portugal [77] and Brazil [78] have up-to-date regional data available regarding this and similar indicators, such as infant, neonatal, and maternal mortality rates, the Angolan database [72,79] is limited to the basic sanitation access rate. Lack of basic sanitation has been broadly described as a major contributor to a larger disease burden and higher mortality rates [80,81,82,83].

Generically, regional indicators, as shown in Table 1, were chosen based on the availability of Angolan data, which limited the resources that could be used for the analysis.

### 2.2. Data Analysis

Mean (± standard deviation) and number (n, %) are used as descriptive statistics. The normality of the data distribution was assessed using the Kolmogorov–Smirnov test with Lilliefors’ significance correction. The homogeneity of variance was assessed using Levene’s test. Welch’s ANOVA followed by pairwise comparisons with the Games–Howell post hoc test (for continuous variables) was used to compare age and mean scores of students’ responses in the ISS4CP dimensions between countries. The chi-squared test was used to examine differences in proportions of distributions regarding general information (e.g., sex and research project) between countries and regions. Linear regression models were performed to predict the attitudes, motivation, skills for clinical practice, and ability of Portuguese, Brazilian and Angolan first-year medical students based on age, sex, school, research project, regional GDP per capita, literacy rate of people aged 15 years and above, basic sanitation access rate, and population density rate.

All assumptions for the statistical procedures were met. Significance level was set at 0.05. All statistical analyses were performed using IBM SPSS Statistics for Windows, Version 21.0 (IBM Corp., Armonk, NY, USA). Ethical principles for this project followed the guidelines approved by FMUP, FMB/Unesp, and FMUKB.

## 3. Results

The sample comprised a total of 372 students: 62.90% from Portugal, 20.16% from Brazil and 16.94% from Angola, the Angolan students being the oldest in first-year of medical school (*p* < 0.001), as shown in Table 2. Of the total Portuguese, Brazilian, and Angolan responding students, 35%, 55%, and 44%, respectively, were male. Regarding secondary level, 29%, 95%, and 15% of the Portuguese, Brazilian, and Angolan students had attended a private school, respectively. Regarding participation in research projects, Portuguese and Angolan students reported 28% and 14% participation, respectively, whereas Brazilian students reported no participation at all.

### 3.1. Comparison of Students’ Attitudes, Motivation, Clinical Practice Skills, and Ability between Portugal, Brazil, and Angola

First-year medical students’ attitudes, motivation, importance attached to scientific skills in clinical practice, and perceived ability to perform them were compared between the three countries. As shown in Table 3, Brazilian students attributed the lowest score to positive attitude (*p* < 0.001) and Angolan students attributed the highest score to negative attitude (*p* < 0.001).

Angolan students achieved the highest score for the integration of undergraduate medical research into the curriculum (*p* < 0.001) and were the most motivated to conduct research during a medical course (*p* < 0.001).

Portuguese students attributed the highest importance to scientific skills for clinical practice, whether they were communication, research, or organization skills, and scored higher when evaluating their own ability to perform those skills (*p* < 0.001). All students, regardless of the country, attributed a higher score to organization skills.

### 3.2. Predictors of Students’ Attitudes, Clinical Practice Skills, and Ability in Portugal, Brazil, and Angola

First-year medical students’ attitudes, importance attached to scientific skills for clinical practice, and perceived ability to perform them were predicted based on age, sex, school, research project, GDP per capita, Population density rate, literacy rate, and basic sanitation access rate (Table 4 and Table 5). Of the variability in Portuguese students’ negative attitudes, 2.6% was explained by GDP per capita, being positively associated (increases 0.000039 points for each EUR 1). Of the variability in Angolan students’ negative attitude toward science and research, 17.9% was explained by school and it significantly increased when they attended private secondary schools (0.526 points) compared to students who attended a public school. Brazilian students’ negative attitudes were not predicted by the variables included in this model.

We found that 6.3% of the variability in the importance attributed by Portuguese students to scientific skills in clinical practice was explained by GDP per capita and sex, being negatively associated with higher GDP (decreases 0.000034 points for EUR 1) and compared to men, women students attributed higher importance to those skills (0.114 points). Of the variability in Angolan students’ perceptions about the importance of skills in clinical practice, 13.9% was explained by research project. Importance significantly increased when students had previously participated in research (0.260 points) compared to those who did not. In the case of Brazilian students, 7.0% of this variability was explained by age, being positively associated (increases 0.036 points for each year).

We found that 4.7% of the variability in Portuguese students’ self-perceived ability to perform scientific skills was explained by both school and research project. This perception significantly increased when they attended private school (0.129 points) and participated in research projects (0.111 points) compared to those who attended public school and did not participate in research, respectively. We were unable to predict Angolan and Brazilian students’ ability. No models were able to be calculated for positive attitude, integration, or motivation.

## 4. Discussion

Our findings offer insight into the attitudes, motivation, and perceptions toward science and research of first-year medical students in three medical schools in Portugal, Brazil, and Angola, and indicate the variations in their predictors between countries. The three countries are at different stages of social, cultural, economic, and demographic development. Portugal is a developed and high-income country, while Brazil and Angola are upper–middle-income countries and considered developing countries [84].

This study’s findings show that Angolan students are the oldest admitted to medical school compared to Brazilians and Portuguese. In Portugal, the prospects of a medical career are widely embraced, and highly ranked students are often, regardless of their vocation, motivated to pursue this career path. The differences observed between schools regarding sex, type of school attendance, and participation in research projects are most likely due to cultural, social, and political differences [38,85]. For instance, regarding sex, a feminization tendency among medical students has been broadly described [85].

Compared to the other students, Brazilians scored lowest regarding positive attitude towards science and scientific research, being also among the least willing to integrate research into the medical curriculum and to perform research during the medical course. Thus, the acquisition of scientific skills did not seem to be viewed as a priority for professional improvement by Brazilian medical students in this study. They are probably unaware of the importance of research training in professional development and clinical practice [45], lacking interest in science, as previously reported by other studies [56].

Angolan students were the most willing to integrate research into medical curriculum and the most motivated to perform research during the medical course. Nonetheless, they reported the most negative attitudes toward science, being among those who attributed less importance to scientific clinical practice skills, apart from communication skills, and were some of the least confident on their own ability to perform them. Recent efforts to expand and develop medical education in Angola appear to be effective among first-year medical students, though they still seem to mistrust science. Although highly motivated toward science and research, welcoming its inclusion into medical curricula [33], they do not seem fully aware of the benefits of scientific research in professional development and clinical practice [56].

Overall, Portuguese students attributed the highest importance to scientific clinical practice skills and were the most confident in their own ability to perform them. Given the high admission GPA in Portuguese medical schools, especially at FMUP, it is not surprising that students feel confident in their abilities. Nonetheless, they were the least motivated to perform scientific research during the medical program, similar to Brazilian students, a finding that might be due to their training, which is theory-based and does not stimulate research training, instead directing students toward clinical practice.

This study’s finding revealed that Angolan students who had attended private schools had more negative attitudes toward science and scientific research compared to those who had attended public schools. Although almost all Angolan students attended a public school, the differences observed between public and private schooling reinforce the idea of a much-needed reliable and equalizing high school educational system, adapted to students’ social, cultural, and environmental backgrounds to better prepare them for the challenges of medical school, while promoting excellence in later professional life [38].

Portuguese students’ negative attitudes toward science are worryingly associated with GDP per capita. The wealthier the regions where students completed high school, the more negative the attitude they disclosed. A change in social paradigm is needed to counter this. Moreover, resources are now scarce, and their use must be enhanced and prioritized to overcome the lack of scientific literacy of medical students.

As previously observed [11,55], in this study, women students also attached higher importance to scientific skills in clinical practice compared to men. For Brazilian students, perceived importance varied according to age, with older students attributing higher importance to research skills, suggesting a more mature and complex approach to the medical profession. Additionally, Portuguese students’ perceived importance of scientific skills differed according to GDP per capita, with students from more deprived regions attaching higher importance to these skills. Angolan students who participated in research projects attributed more importance to scientific skills in clinical practice, reinforcing the relevance of research activities for students’ professional development. Greater financial support to these projects is imperative to overcome the limiting social and financial barriers to participation in research [38,39].

Portuguese students’ self-reported ability to perform scientific skills differed according to the type of secondary school they attended and previous participation in research. Students who attended private schools self-evaluated their preparedness as higher than those who attended public schools. Similarly, participation in research projects also improved students’ confidence on their abilities to perform scientific skills, as supported by other available evidence [55]. As self-confidence in scientific skills was reported to be associated with personal interest in research, the prospects of positive results, and higher academic achievement, medical educators are now responsible for disclosing and promoting research opportunities and efficiently involve students in meaningful learning experiences [28,55].

Despite regional social, cultural, environmental, and economic disparities, especially in Angola and Brazil, none of the indicators, such as regional rates of population density, literacy, or basic sanitation access, affected first-year medical students’ attitudes or perceptions. These findings might be explained by the inclusion of only one medical school from each country, thereby highly limiting the sample, despite the different regional backgrounds of the students and, in the case of Angola and Brazil, the relatively small sample size compared to Portugal. Furthermore, the efforts made by the three medical schools to minimize educational differences among students are probably expressed in this study’s results.

Self-perceived ability is an element of self-efficacy [86] and reflects the motivation to continuously develop specific competences, identifying areas for improvement. Thus, proper assessment of students’ perceptions regarding their education can validly be used to measure the quality of medical education itself [7,87].

### Limitations

Despite the contributions of this study, some limitations can be identified: Data collection from the three medical schools occurred at different times. The nonrandomized design was the only one possible, as the whole population of first-year medical students from the participating schools was selected. Despite adjustments for different educational, economic, geodemographic, and public-health backgrounds, the inclusion of only one school from each country might have compromised the external validity of the study. Additionally, the schools differed in the number of students. The legal administrative divisions for each country, aside from being used in the regional distribution of the indicators, were intended to be used to compare the different regions and assess differences as well. However, due to the limited sample size, exacerbated by further divisions, it was not possible to investigate differences between the regions. The ISS4CP questionnaire was applied to non-Portuguese populations for the first time, but the scale revealed adequate internal consistency. Finally, the lack of anonymity might have led to social desirability bias, where students tried to favorably describe themselves.

## 5. Conclusions

This study’s findings revealed differences in the attitudes, motivations, and perceptions toward science and research of first-year medical students in Portugal, Brazil, and Angola. For each country, factors influencing students’ attitudes and perceptions also varied, mostly predicted by individual characteristics (sex, age, participation in research, and type secondary school attended); of the regional indicators assessed, only GDP per capita emerged as a significant predictor.

These results add to the existing literature on the topic, which is particularly scarce in low-income countries, such as Angola and Brazil. They offer useful information for educational and political stakeholders, contributing to informing the integration of tailored research programs in medical schools, adjusted to different local needs and contextual challenges and demands. This study also provided a valuable opportunity to exchange knowledge and experience, promoting mutual learning and collaboration between countries.

Our findings contribute to guiding future cross-cultural research that can expand our design to include more medical schools in different country regions, as well as further explore these and other predictors of students’ attitudes and perceptions toward science and research. Additionally, follow-up studies can provide more accurate information regarding the design and implementation of best-adjusted undergraduate medical research programs, aiming to increase the numbers of physician-scientists, thus benefiting patient health care.

## Figures and Tables

**Table 1:** Summary of regional indicators.

Country	Region	GDP per Capita (Euros)	Population Density (No. Inhabitants/km^2^)	Literacy Rate of People ≥ 15 Years Old (%)	Basic Sanitation Access Rate (%)
Portugal	North w/out GP	13,548.000 ***	173.339 ****	89.70 ****	100.00 *
	Great Porto	16,400.000 ****	1580.302 ****	90.00 ****	100.00 *
	Centre	14,165.000 ****	82.546 ****	87.00 ****	100.00 *
	Madeira RA	16,412.000 ****	334.272 ****	87.00 ****	100.00 *
	Azores RA	15,226.000 ****	106.276 ****	89.50 ****	100.00 *
Brazil	São Paulo	33,624.410 *****	168.805 ***	85.12 ***	100.00 **
	Minas Gerais	20,324.580 *****	33.853 ***	83.44 ***	92.00 **
	Goiás	20,134.260 *****	18.097 ***	82.87 ***	28.00 **
Angola	Luanda	12,037.239 *	2898.956 ******	86.70 ******	89.80 ****
	Huila	306.855 *	31.391 ******	67.90 ******	46.00 ****
	Benguela	6017.317 *	64.070 ******	56.10 ******	34.80 ****

* data from 2007; ** data from 2008; *** data from 2010; **** data from 2011; ***** data from 2012; ****** data from 2014.

**Table 2:** Comparison of general information between Portugal, Brazil, and Angola.

			Country		
		Portugal	Brazil	Angola	*p*-Value
Age, M (SD)		19.07 (4.826)	19.96 (2.121)	22.48 (6.459) a	<0.001
Sex, *n* (%)	Male	82 (35.0)	41 (54.7)	28 (44.4)	0.008
	Female	152 (65.0)	34 (45.3)	35 (55.6)	
School, *n* (%)	Private	67 (29.1)	71 (94.7)	9 (15.0)	<0.001
	Public	163 (70.9)	4 (5.3)	51 (85.0)	
Research Project, *n* (%)	Yes	66 (28.2)	0	7 (13.7)	<0.001
	No	168 (71.8)	75 (100)	44 (86.3)	

Post hoc analysis: (a) Angola is different from Portugal (*p* = 0.001) and Brazil (*p* = 0.013). M—mean; SD—standard deviation.

**Table 3:** Comparison of attitudes, motivation, skills, and ability between Portugal, Brazil, and Angola.

		Country			
Domain		Portugal (*n* = 234)	Brazil (*n* = 75)	Angola (*n* = 63)	*p*-Value
**Attitudes** M (SD)	Positive	3.374 (0.311)	3.005 (0.305) ^a^	3.314 (0.336)	<0.001
	Negative	1.985 (0.312)	1.943 (0.281)	2.362 (0.447) ^b^	<0.001
	Integration	3.140 (0.331)	3.030 (0.374)	3.319 (0.337) ^c^	<0.001
**Motivation** M (SD)		2.774 (0.597)	2.800 (0.689)	3.152 (0.499) ^d^	<0.001
**Clinical** **Practice Skills** M (SD)	Communication	3.484 (0.410) ^e^	3.035 (0.579)	3.219 (0.571)	<0.001
	Research	3.314 (0.416) ^f^	3.125 (0.538)	2.979 (0.570)	<0.001
	Organization	3.529 (0.422) ^g^	3.360 (0.500)	3.236 (0.585)	0.001
**Ability** M (SD)		3.439 (0.348) ^h^	3.186 (0.444)	3.138 (0.513)	<0.001

Post hoc analysis: (^a^) Brazil is different from Portugal (*p* < 0.001) and Angola (*p* < 0.001); (^b^) Angola is different from Portugal (*p* < 0.001) and Brazil (*p* < 0.001); (^c^) Angola is different from Portugal (*p* < 0.001) and Brazil (*p* < 0.001); (^d^) Angola is different from Portugal (*p* < 0.001) and Brazil (*p* = 0.001); (^e^) Portugal is different from Brazil (*p* < 0.001) and Angola (*p* = 0.002); (^f^) Portugal is different from Brazil (*p* = 0.016) and Angola (*p* < 0.001); (^g^) Portugal is different from Angola (*p* = 0.002); (^h^) Portugal is different from Brazil (*p* < 0.001) and Angola (*p* = 0.001). M—mean; SD—standard deviation.

**Table 4:** Multiple linear regression model of the association between negative attitude, clinical practice skills, and ability and age, sex, school, research project, GDP per capita, population density rate, literacy rate, and basic sanitation access rate by country.

Domain	Country	Model		Sum of Squares	dF	F	*p*-Value
Negative Attitude	Portugal	a	Regression	0.530	1	5.537	0.020
			Residual	19.702	206		
			Total	20.231	207		
	Angola	b	Regression	1.412	1	8.297	0.006
			Residual	6.466	38		
			Total	7.878	39		
Clinical Practice Skills	Portugal	c	Regression	0.691	1	8.672	0.004
			Residual	16.809	211		
			Total	17.500	212		
		d	Regression	1.094	1	7.003	0.001
			Residual	16.406	210		
			Total	17.500	212		
	Angola	e	Regression	0.344	1	6.145	0.018
			Residual	2.129	38		
			Total	2.473	39		
	Brazil	f	Regression	0.374	1	4.737	0.033
			Residual	4.968	63		
			Total	5.342	64		
Ability	Portugal	g	Regression	0.630	1	5.036	0.026
			Residual	26.657	213		
			Total	27.287	214		
		h	Regression	1.167	2	4.734	0.010
			Residual	26.121	212		
			Total	27.287	214		

Predictors: (a) (constant), GDP per capita; (b) (constant), school; (c) (constant), sex; (d) (constant), sex and GDP per capita; (e) (constant), research project; (f) (constant), age; (g) (constant), school; (h) (constant), school and research project.

**Table 5:** Selected multiple linear regression model of association between negative attitude, clinical practice skills, and ability, and age, sex, school, research project, GDP per capita, population density rate, literacy rate, and basic sanitation access rate for each country.

Domain	Country	Model	b	β	R^2^	Adjusted R^2^	sr^2^	*p*-Value
Negative Attitude	Portugal	Constant	1.402		0.026	0.021		<0.001
		GDP per capita	0.000039	0.162			0.026	0.020
	Angola	Constant	2.238		0.179	0.158		<0.001
		School	0.526	0.423			0.179	0.006
Clinical Practice Skills	Portugal	Constant	4.089		0.063	0.054		<0.001
		Sex	−0.114	−0.189			0.038	0.005
		GDP per capita	−0.000034	−0.152			0.025	0.024
	Angola	Constant	3.642		0.139	0.117		<0.001
		Research Project	0.260	0.373			0.139	0.018
	Brazil	Constant	2.738		0.070	0.055		<0.001
		Age	0.036	0.264			0.070	0.033
Ability	Portugal	Constant	3.359		0.047	0.034		<0.001
		School	0.129	0.163			0.027	0.017
		Research Project	0.111	0.141			0.020	0.038

Note: sr^2^ is the squared semi-partial correlation.

## Data Availability

The data presented in this study are available on request from the corresponding author.
